# On the Treatment of Inflammation by Digital Compression

**Published:** 1859-11

**Authors:** M. Vanzetti

**Affiliations:** Professor of Clinical Surgery in the University of Padua


*On the Treatment of Inflammation by Digital Compression*, by M. Vanzetti, Professor of Clinical Surgery in the University of Padua. (From the Giornale Veneto di Scienze Mediche, l'Union Médicale, Dec. 30th, 1858.)

We have already (Jan. 1859) recorded the results of two cases in which M. Vanzetti successfully practiced digital compression in inflammation; and the following cases appear to establish the advantage derivable from this method of treatment:—

The first case was one of phlegmonous inflammation of the right leg, occurring in a man, aged thirty-eight. When he was admitted into the hospital, the right leg was red and considerably swollen throughout its lower half as far as the metatarsal bones; the redness was most intense over the internal ankle, which appeared to be the most painful part, and the center from which the inflammation extended to the rest of the limb. The pain was increased on pressure, especially at the internal ankle, and the finger left a well-marked depression on the leg in consequence of the acute œdema of the subcutaneous areolar tissue. Compression was immediately commenced—namely, at seven o'clock in the evening; and the pulsations of the femoral artery, which was compressed at the ilio-pectineal eminence, were so violent that it was necessary to employ some force in order completely to interrupt the circulation. M. Vanzetti saw the patient at eight o'clock, and found that the black thread, with which the limb had been surrounded, was already relaxed; the redness was much less intense, the pain had almost entirely ceased, and the patient said that he felt remarkably relieved. The compression was continued during the whole of the night, with some short interruptions; and on visiting the patient the next day, at half-past ten in the morning, a surprising change was found to have taken place in the state of the affected limb, which no longer presented any of the symptoms of phlegmon which had been so distinct on the previous evening; and it was therefore considered unnecessary to continue the compression. In order to assure himself that the affected parts had returned to their normal state, M. Vanzetti caused the patient to rise and walk several times along the ward; he did not complain of the slightest pain in walking, and he refused the aid of a stick which was offered to him. The man recovered completely, and on the third day after the commencement of the treatment he returned to his work.

The second case was one of traumatic phlegmon of the left hand, occurring in a girl of fourteen, who, having had a fall while holding a bottle, received a wound in the palm of the left hand from a fragment of glass. On the sixth day after the accident, fever supervened, and acute pain was felt in the hand; and the next day the girl came to the hospital. The hand then exhibited, especially on its dorsal surface, an intense redness, with considerable swelling, and pain on pressure, and the inflammation extended to the whole of the circumference of the forearm. The thumb and index finger were also swollen—red, tense, and shining; the pulse was 108. Compression of the humeral artery was now resorted to, the pulsations of the radial artery at the wrist indicating the exact degree of the compression; but the patient was also able to explain whether the process was well or badly performed by the symptoms which she experienced. After only two hours the compression had produced a sensible amelioration; the thread which had been tied over the most projecting part of the hand already allowed the little finger to pass beneath it, and the heat and redness had diminished. In six hours afterwards the symptoms had diminished still more; the compression was continued without preventing the patient from sleeping, and the subclavian artery was occasionally compressed. On the next day the redness was found to be circumscribed to the back of the hand; the index finger was now easily passed beneath the thread employed to measure the size of the limb; the heat was less, and the pain had entirely ceased. The pulse was 100. Two days afterward no trace of inflammation remained; but it was now ascertained that a small fragment of glass was imbedded in the tissues of the hand. It was extracted without difficulty, and in a week more the patient was quite well.

The third case was one of diffuse phlegmon of the left leg, occurring in a man, aged fifty-six. There was considerable swelling of the whole of the limb, the circumference of which exceeded that of the sound limb by three inches; the tissues were tense and elastic, but there was no œdema, except at the middle portion of the anterior surface of the tibia, which was also the seat of a very acute pain on pressure. Compression was practiced; and during the first few hours which followed the adoption of this measure the patient already experienced considerable relief, and he said that he no longer felt the sensation of heat by which he was previously so much annoyed. Compression having been commenced at eight o'clock in the morning, there was so much amelioration at four o'clock in the afternoon that the index finger could be passed under the thread employed to measure the size of the leg. At nine in the evening the improvement still continued. During the night the compression was continued, but with short intervals; and the patient, satisfied with the relief afforded by the process, performed it himself, but interrupting it from time to time in order to sleep. The next day at ten o'clock in the morning, all the symptoms of the disease had entirely ceased; there was no longer any swelling or tension of the tissues. The pulse was 56. The patient could walk without difficulty or pain; and after remaining six days in the hospital, he went away perfectly cured.—*Ibid.*

